# Time for a drought experiment: Do you know your plants’ water status?

**DOI:** 10.1093/plcell/koac324

**Published:** 2022-11-08

**Authors:** Thomas E Juenger, Paul E Verslues

**Affiliations:** Department of Integrative Biology, University of Texas, Austin, Texas 78712, USA; Institute of Plant and Microbial Biology, Academia Sinica, Taipei 11528, Taiwan

## Abstract

Drought stress is an increasing concern because of climate change and increasing demands on water for agriculture. There are still many unknowns about how plants sense and respond to water limitation, including which genes and cellular mechanisms are impactful for ecology and crop improvement in drought-prone environments. A better understanding of plant drought resistance will require integration of several research disciplines. A common set of parameters to describe plant water status and quantify drought severity can enhance data interpretation and research integration across the research disciplines involved in understanding drought resistance and would be especially useful in integrating the flood of genomic data being generated in drought studies. Water potential (ψ_w_) is a physical measure of the free energy status of water that, along with related physiological measurements, allows unambiguous description of plant water status that can apply across various soil types and environmental conditions. ψ_w_ and related physiological parameters can be measured with relatively modest investment in equipment and effort. Thus, we propose that increased use of ψ_w_ as a fundamental descriptor of plant water status can enhance the insight gained from many drought-related experiments and facilitate data integration and sharing across laboratories and research disciplines.

## Teaser

Drought stress experiments are difficult to conduct and interpret. We highlight how integrating measurements of water status, particularly water potential, with genetic and genomic assays would benefit many types of plant stress research.

## Introduction

Drought is a topic of strong interest across plant biology from agronomy to ecology and to molecular and cell biology. The prospect that climate change will cause more frequent drought episodes will only add to this interest in the coming years. From an agronomic perspective, the goal of many studies is to identify and study factors that influence crop yield and how these factors are modified by drought. From the ecological perspective, there are strong interests to understand how plants adapt to water-limited environments and how that adaptation scales to community and ecosystem function. Molecular genetics research aims to find the genes and cellular mechanisms plants use to detect and respond to drought stress and thereby develop fundamental knowledge of plant function. A common thread of these types of plant drought research is that they seek to understand the genotype by environment interactions that determine the distribution and productivity of different plant species. All these research fields also have a strong interest in being able to make predictions and interventions: predictions of how climate change will alter ecosystems or crop productivity; and, interventions, such as genetic modification of crop plants or ecosystem management strategies, that can mitigate the effects of drought.

These objectives require scaling across different disciplines and types of experiments. Multiple types of scientists need to be able to compare their studies of drought stress and learn from each other’s results. This in turn requires a common terminology and core methods to quantitate the severity of drought stress and report the most relevant plant responses. Then researchers can get the most insights from their data and have confidence in comparing the results of different studies. But, what are the best methods to quantify drought stress severity? Salt stress can be reported as salt concentration or changes in the conductivity of soil water. Temperature stresses (freezing, chilling, and heat stress) can be standardized based on the timing and extent of the temperature change (and whether ice nucleation is provided in the case of freezing) (Verslues et al., 2006). In this article, we describe equivalent measures for “drought” stress and show how incorporating such measurements can increase the insight gained from many types of experiments, including ‘omics approaches, and also allow us to avoid common pitfalls in data interpretation.

The above sentence refers to “drought” in quotations because some readers may point out, correctly, that the core definition of drought is meteorological (a period of below-average precipitation). However, for much of plant biology, and the majority of papers in this journal, the term drought is instead used to refer to the plant’s relationship with water (i.e. plant water status). Thus, “drought” responses as commonly discussed in most molecular and cellular studies are really responses to an altered water status where water has become less available to the plant because it is at a lower free energy state compared with unstressed conditions. For plant biology, the energy state of water can be quantified and reported in terms of the water potential (ψ_w_).

## ψ_w_ and water movement through the SPAC: A brief primer on plant water relations

**Figure 1:**
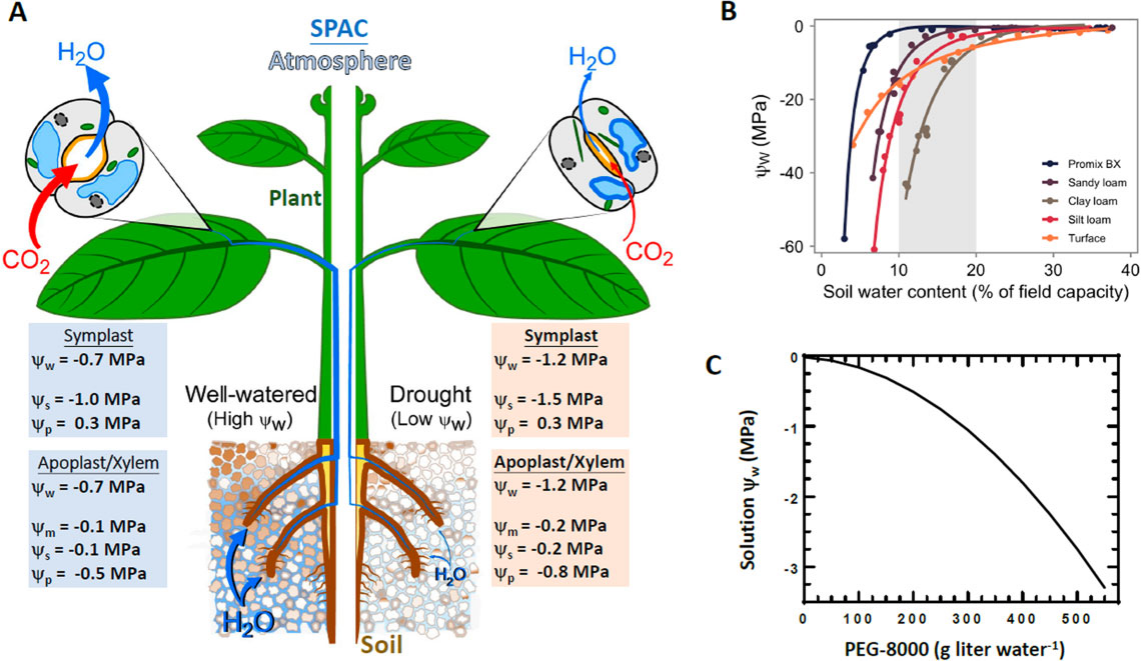
Water movement through the SPAC and water status of soils, solutions and plant tissue can be described in a unified manner by ψ_w_. A, Diagram of water movement through the SPAC. A continuous column of water exists from the soil, through the root tissue and into the xylem, and into capillary spaces around leaf cells. Evaporation of water from leaf tissue and adhesion of water molecules pulls water up through the SPAC. The rate of water evaporation from leaf tissue is controlled primarily by the opening and closing of stomata. The boxes give typical values of ψ_w_ and its components inside plant cells (symplast) or just outside these cells (apoplast/xylem) for well-watered conditions or drought conditions. The low ψ_w_ conditions shown would be within the range experienced by crop plants (or other mesophytic plants) and would be at or just above the permanent wilting point (i.e. the ψ_w_ below which the plant cannot regain turgor even overnight when stomata close and transpiration is minimal) for most such species (depending on light, temperature and air humidity conditions). B, Example soil moisture retention curves for five different soils highlighting the potential variability in soil ψ_w_ (*x*-axis; Ψ_W_) at various soil–water contents (*y*-axis; expressed as a percentage of field capacity). The banded area (10–20% of field capacity) highlights the range of soil–water content typically utilized in studies imposing drought stress on plants. Data were collected using the Decagon WP4 Dewpoint Potentiometer. Curves are presented for two common horticultural mixes (Promix BX, Turface) along with several field soils spanning a gradient of texture and composition (sandy loam, clay loam, and silt loam). For plotting purposes, the best fitting model (second-order polynomial of the reciprocal term and soil interaction) was selected by Akaike Information Criterion and showed an adjusted *R*^2^ of 0.9893 (*P* <1*e*−10) and significant differences among soils. All statistical analyses were performed with the stats package in the R software environment (R Development Core Team). C, Relationship between PEG-8000 concentration and ψ_w_. Note that the values are for a given amount of PEG-8000 added to 1 L of water without adjustment of the final volume (a significant increase in volume will occur for higher PEG concentrations). The ψ_w_ of PEG solutions was measured using a Wescor Psypro system with C52 sample chambers following the manufacturer’s instructions and using ψ_w_ standards provided by the manufacturer (Verslues, 2010) or by using a Wescor vapor pressure osmometer. For the osmometer, osmolality readings in mmol kg^−1^ were converted to ψ_w_ using the van’t Hoff calculation (Verslues, 2010; Banks and Hirons, 2019). Both instruments gave essentially identical results. Note that because PEG-8000 is a large hygroscopic molecule, which affects ψ_w_ primarily by adherence to water molecules, there is a nonlinear relationship between PEG content and ψ_w_, similar to the nonlinear relationship between soil ψ_w_ and water content. Data are replotted from Verslues et al. (2006) and from previously unpublished data of the Verslues laboratory.

Land plants move copious quantities of water through the soil–plant–atmosphere continuum (SPAC) whereby water taken up from the soil moves upward through the plant vascular system (Figure 1A). Most of this water is lost from the plant by transpiration through stomata. The movement of water through the SPAC is driven by differences in ψ_w_. The ψ_w_ is defined as the amount by which the chemical potential of water is reduced below that of pure water (pure water being zero, thus ψ_w_ is always negative) and is expressed in units of pressure (Kramer and Boyer, 1995). A higher concentration of dissolved solutes will decrease osmotic potential (ψ_s_) to lower (more negative) values. Adhesion of water molecules to surfaces also decreases their free energy; this is referred to as the matric potential (ψ_m_) and is an important determinant of soil ψ_w_. However, as most techniques for measuring ψ_w_ cannot separate the effects of solutes versus adherence of water molecules along surfaces (e.g. the surface of soil particles), ψ_m_ is often considered to be part of ψ_s_ for practical purposes. A positive pressure (ψ_p_), in turgid cells, for example, will increase ψ_w_ (make it less negative) while a negative pressure (e.g. in xylem as water is pulled up by transpiration) will reduce ψ_w_. A higher position in gravity (ψ_g_) will also make ψ_w_ more negative; but, the effect of gravity is negligible for those not studying large trees and is also typically omitted for practical purposes. Thus, for purposes of most plant biology research, ψ_w_ is essentially determined by two major components, osmotic potential ψ_s_ (which is denoted as π in some cases, also referred to as “solute potential”) and ψ_p_. Thus, the full set of ψ_w_ components (ψ_w_ = ψ_s_ + ψ_m_ + ψ_p_ + ψ_g_) can be reduced to the basic ψ_w_ equation of ψ_w_ = ψ_s_ + ψ_p_ (Kramer and Boyer, 1995).

At different points in the SPAC, the components of ψ_w_ differ. In the soil, matric forces of water adhering to soil particles are the dominant component of ψ_w_ (except in highly saline soils where dissolved salts also decrease ψ_s_). Inside the plasma membrane of plant cells, the active accumulation of solutes decreases ψ_s_ to drive water uptake. This osmotically driven uptake of water produces a positive turgor pressure inside plant cells because they have rigid cell walls which restrict cellular volume. As solutes accumulate, turgor pressure will increase until the cell reaches ψ_w_ equilibrium with its immediate surroundings (Figure 1A). In wall-less cells, the accumulation of solutes determines the cell volume. Thus, all cells must osmoregulate (regulate their solute content) at all times, to maintain cell volume or regulate turgor. In the apoplast and xylem, tension (negative pressure) generated by adhesion of water molecules moving up through the plant (and some matric and solute effects) is the major component of ψ_w_. This upward movement of water is driven by water evaporation from capillary surfaces in the inner air spaces of the leaf. As water vapor, even at near saturation humidities, has a much lower ψ_w_ than liquid water, evaporation can always occur at appreciable rates. Thus, the rate of water loss from the leaf is controlled primarily by stomata. The regulation of stomatal opening and closing, as well as control of stomatal size and density, is key to balancing the competing priorities of controlling water loss while allowing CO_2_ uptake.

For readers from a plant physiology background, the above information is well known. For those approaching plant stress biology from other backgrounds who are interested in a more thorough introduction to plant water relations, we recommend the Taiz and Zeiger *Plant Physiology* textbook (renamed as *Plant Physiology and Development* for later editions) (Taiz and Zeiger, 2006) as well as the comprehensive book of Kramer and Boyer (1995) or its methods focused companion (Boyer, 1995) or, the following web pages provided by makers of instruments for ψ_w_ measurement (Ψ_w_ versus water content).

## Advantages of monitoring ψ_w_ in plant biology research

A key advantage of ψ_w_ for plant research, compared with other metrics such as soil water content, is that it allows laboratory or field-based stress treatments to be described in a manner that is unambiguous and thus able to be replicated across experimental systems while also giving a clearer picture of the stress severity experienced by the plant. The different distribution of particle sizes among soil types means that the relationship between soil water content versus ψ_w_ varies greatly among soil types (Figure 1B). Clay soils (smaller average particle size) have much higher water content at even very low ψ_w_ than sandy soils which have larger average particle size and thus less surface area for water adhesion (Kramer and Boyer, 1995); see also Soil water content versus ψ_w_). Thus, a drought stress severity reported in terms of soil water content could not be reproduced in another laboratory by subjecting plants to the same soil water content unless both laboratories used exactly the same soil. Many soils, including the peat-based horticultural soil mixes often used in plant research, exhibit a dramatic decline in soil ψ_w_ once soil water content passes a threshold level (Figure 1B; Walczak et al., 2002; Fields et al., 2014; Dowd et al., 2019). Thus, a seemingly small difference in soil water content between different replicates or different genotypes can actually indicate a substantial difference in stress severity. This is most acute for sandy soils (soils comprised mostly of large particles with lower portion of smaller silt and clay-type particles), as these soils have low water-holding capacity.

In some studies, calculation of the fraction of transpirable soil water (FTSW) has been used to estimate the degree of water limitation experienced by plants. FTSW utilizes soil moisture data along with the threshold soil water content (or threshold ψ_w_) below which the plant under study can no longer extract water (i.e. transpiration decreases to near zero) to calculate the relative amount of plant available water that remains at various bulk soil water contents (Serraj and Sinclair, 1997). Since the threshold for water extraction is determined largely by ψ_w_ (along with soil hydraulic conductivity), FTSW is strongly correlated with soil ψ_w_ or leaf ψ_w_ (see e.g. Lacape et al., 1998; Yan et al., 2017; Devi and Reddy, 2020). FTSW can be useful for field studies and irrigation scheduling; however, for laboratory or greenhouse studies, measurement of ψ_w_ still offers a more straightforward measure of stress severity that is physically grounded and not dependent upon the properties of the growth media or properties of the plants under study. Use of ψ_w_ also facilitates comparison of results between soil experiments and experiments conducted in other types of media where FTSW is not applicable, such as agar plates or liquid culture. It should also be noted that in studies of plant stress acclimation, it has been observed that many important physiological parameters, such as abscisic acid (ABA) accumulation, proline accumulation, and growth show a linear or near-linear relationship with ψ_w_ (see e.g. van der Weele et al., 2000; Verslues and Bray, 2004; Liu et al., 2005) . Use of ψ_w_ to scale data is further discussed later in this article. Thus, for genetic studies of drought response, FTSW may be a supplement to, but not a replacement for, measurement of ψ_w_.

For such laboratory- or greenhouse-based studies of controlled soil drying, where the type of growth media used can be deliberately selected, the approach described by Dowd et al. (2019) may be applicable. They analyzed several types of growth media and found dramatic differences in water-holding capacity. They then selected the type of growth media that had a high water-holding capacity over the range of low-to-moderate ψ_w_ treatments they wished to impose (−0.25 to −0.4 MPa; the unstressed control was −0.1 MPa). By using this selection of appropriate growth media, along with monitoring of soil weight and maintaining a high humidity around the soil surface, they were able to expose maize seedlings to stable ψ_w_ treatments over 9 days to quantify the effects of ψ_w_ on maize lateral root development (Dowd et al., 2019). Thus, they could assay effects of reduced ψ_w_ on root development that would not be apparent if they used a media with low water holding capacity over the target range of ψ_w_ and not apparent if they had simply allowed the soil to dry rapidly over the experimental period.

It is also important to note that declines in soil ψ_w_ lead to a dramatic decline in soil hydraulic conductivity (i.e. the water that remains in the soil is more tightly bound to soil particles). This decline in hydraulic conductivity varies greatly among soils and determines how much water the soil can supply to the plant at a given ψ_w_. Soil hydraulic conductivity can be a key factor in determining stomatal opening (and perhaps other drought-responsive traits) as the plant seeks to match the water supply available from the soil with transpirational demand (Carminati and Javaux, 2020).

For other types of experiments, high molecular weight polyethylene glycol (PEG; such as PEG-8000) is a useful agent to impose low ψ_w_ upon plants, especially when the PEG is incorporated into a solid matrix such as agar plates (van der Weele et al., 2000; Verslues et al., 2006). High molecular weight PEG is useful because large PEG molecules reduce ψ_w_ primarily by matric forces rather than osmotic forces and because large PEG molecules are not able to penetrate the pores of plant cell walls and thus cause cytorrhysis (withdrawal of water from and shrinkage of both cell wall and protoplast) rather than plasmolysis where only the protoplast loses water and may separate from the cell wall (Oertli, 1985). For these reasons, treatment with high molecular weight PEG is more similar to soil drying than osmotic stress imposed with low molecular weight solutes. However, high molecular weight PEG does not behave as an ideal solute and there is a nonlinear relationship between PEG concentration and ψ_w_ (Figure 1C) which needs to be accounted for when determining the amount of PEG needed to impose a moderate versus more severe low ψ_w_ treatment (van der Weele et al., 2000; Verslues et al., 2006). When using PEG for liquid cultures, it should also be kept in mind that solutions of high molecular weight PEG are viscous and can cause root hypoxia unless the solutions are well aerated (Verslues et al., 1998). At the same time, root damage should be avoided as this may allow PEG molecules to enter the plant tissue. Use of PEG-infused agar plates avoids these problems and is a good experimental system for subjecting small plants/seedlings to a constant and defined severity of low ψ_w_ stress (Van der Weele et al., 2000; Verslues et al., 2006) while avoiding complications that may arise from using low molecular weight solutes such as mannitol which, in addition to causing plasmolysis, may elicit specific responses unrelated to changes in ψ_w_ (Trontin et al., 2014).

## Plants respond to restricted water supply by avoiding water loss and tolerating reduced ψ_w_

The key factor in our plant physiological definition of drought is the decline in external ψ_w_. This is primarily caused by reduced soil water content such that the remaining soil water is held by stronger matric forces. Increased solute content of soil water can also be a factor, as well as a high vapor pressure deficit resulting from drying atmospheric conditions (low humidity and high temperature, perhaps accompanied by rapid air movement) which may cause drying of the plant tissue even when soil moisture is still available. The most basic effect of decreased external ψ_w_ is to collapse, or reverse, the ψ_w_ gradient that had allowed the plant tissue to take up water. Thus, when the external ψ_w_ decreases, ψ_w_ of the plant tissue will also decline. If the plant does nothing, this will occur passively by water efflux from cells leading to loss of turgor and decrease in cell volume until equilibrium with external ψ_w_ is reached. Because water flux through the SPAC is rapid, the most immediate need of the plant is to stop the bleeding (of water) by closing stomata. Stomatal closure, along with other drought responses that aim to reduce water loss (e.g. rolling or shedding of leaves, thicker cuticle, increased trichome density, or reduced stomatal density on newly formed leaves) are classically referred to as avoidance responses because they aim to avoid depletion of the available water. For mesophytic plants which genetically prioritize high photosynthesis rates and rapid growth (such as most crop plants), or in cases where soil drying is rapid, avoidance is a key component of the drought response. It is also the dominating factor in many laboratory experiments where plants are grown in a small volume of soil such that terminal drying rapidly occurs once water is withheld from the plant. For these reasons, avoidance responses are the aspects of drought response that we are most familiar with at the molecular genetic level. The stomatal closure responses described above are regulated through relatively well-characterized signaling pathways involving ABA and other plant hormones (see e.g. Vaidya et al., 2019; Yang et al., 2019; Berrio et al., 2022).

**Figure 2:**
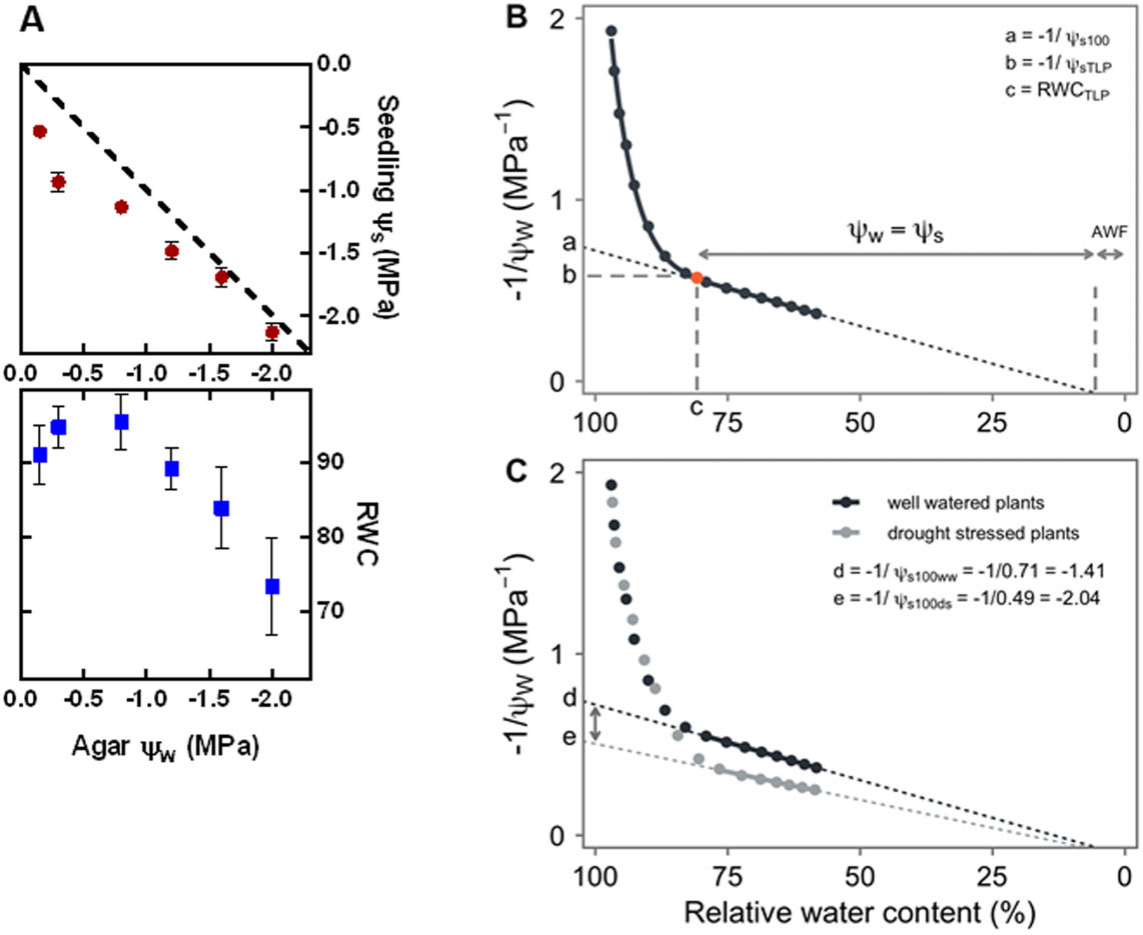
Quantifying solute content, turgor, and plant tissue water content. A, Seedling osmotic potential (ψ_s_) and RWC of *A. thaliana* (Col-0 accession) seedlings provide a simple illustration of osmotic adjustment and the TLP concept (replotted from Verslues, 2010). Seven-day-old seedlings (ecotype Col-0) were transferred to PEG-agar plates of the indicated ψ_w_ and whole seedlings (10–30 per sample, depending on treatment) collected 3 days after transfer. As transpiration is minimal in this system, one can assume that the ψ_w_ of the plant tissue is in near equilibrium with the agar ψ_w_. Thus, a seedling ψ_s_ below the dashed line indicates a positive turgor pressure. When osmotic adjustment can no longer maintain turgor (at ∼−1.2 MPa), further declines in seedling ψ_w_ occur via water loss (indicated by reduced RWC) and passive concentration of solutes. B, A theoretical PV curve representing the expected relationship between −1/ψ_w_ and RWC for a drying leaf. At high RWC, ψ_w-leaf_ is a function of both turgor pressure ψ_p_ and osmotic potential ψ_s_ and exhibits an exponential decline with drying. The TLP (the red dot) occurs when dehydration proceeds until ψ_p_ = 0. The linear decline in −1/ψ_w_ past TLP is driven by the passive concentration of solutes with water loss. The linear extension of the relationship to the *y*-axis reveals the osmotic potential at full hydration (a: −1/ψ_s100_). The osmotic potential at zero turgor (b: −1/ψ_sTLP_) can be determined by extending a horizontal line from TLP to the *y*-axis while the RWC at TLP (c: RWC_TLP_) can be determined by a perpendicular line from TLP to the *x*-axis. Symplastic and apoplastic (AWF) fractions can be inferred from the linear intersection of the fit line with the *x*-axis. The modulus of elasticity (*ε*) can be inferred from the slope of the ψ_p_ from full hydration to the TLP. C, Example PV curves derived from well-water and drought-stressed plants depicting osmotic adjustment and a shift in TLP. The osmotic potential at full turgor for the drought-stressed plant material (e: −1/ψ_s100ds_) can be subtracted from the osmotic potential at full turgor for the well-water watered plant material (d: −1/ψ_s100ww_) to obtain an estimate of osmotic adjustment. C, Redrawn from Sanders and Arndt (2012), adapted by permission from Springer Nature.

While avoidance responses are essential to conserve water and may help slow the rate of soil ψ_w_ decline, this avoidance of water loss cannot itself restore water uptake and turgor. To do this, the plant must decrease its internal ψ_w_ to a value below the external soil ψ_w_ by osmotic adjustment, the active accumulation of additional solutes inside cells (Figure 1A). Osmotic adjustment to maintain turgor is a prerequisite for longer term developmental responses such as increasing the root-to-shoot ratio and changing root growth patterns through maintenance of root elongation to reach deeper in the soil profile, hydrotrophic responses, or altered lateral root initiation. Active osmotic adjustment, as opposed to passive increase in solute content by tissue dehydration, is an adaptive tolerance response as it allows the plant to maintain function at reduced ψ_w_. A simple example of osmotic adjustment in *Arabidopsis thaliana* is shown in Figure 2A where seedlings were transferred to agar plates which had varying amounts of PEG added to generate a range of ψ_w_ from mild stress (small decrease of ψ_w_) that had no apparent detrimental effect to severe stress levels. Seedling ψ_s_ and relative water content (RWC; water content of the tissue relative to its water content when fully hydrated) were measured three days after transfer (Verslues, 2010). The plants were able to fully osmotically adjust and maintain high RWC after transfer to ψ_w_ as low as −1.0 MPa (and because ψ_s_ of the plant tissue is less than ψ_w_ of the agar media, it can be inferred that turgor was also maintained over this range). The extent of osmotic adjustment, and its role in maintaining high RWC even at substantially reduced ψ_w_, can be easily observed in this experimental system because transpiration is minimal and thus avoidance responses do not dominate the phenotype observations in the way that often occurs in pot-based soil drying experiments.

While osmotic adjustment is a fundamental aspect of plant responses to low ψ_w_, whether or not increased osmotic adjustment is of value for improving crop productivity during drought has been controversial. It has been argued that at relatively low ψ_w_ near the permanent wilting point (around −1.5 MPa for most crop plants), the amount of water that could be extracted by osmotic adjustment is likely too small to affect productivity (Morgan, 1995, 2000; Serraj and Sinclair, 2002). At higher soil ψ_w_, increased osmotic adjustment may lead to more rapid water depletion and thus may not be beneficial under prolonged drought where water conservation and increased water use efficiency (WUE) would be more valuable. However, others have strongly disagreed with this assessment, and have argued that the capacity for osmotic adjustment is associated with crop productivity during drought and that root osmotic adjustment in particular can facilitate further growth to reach water in deeper soil layers (Blum, 2017). It seems likely that whether or not increased osmotic adjustment will allow improved plant productivity depends on the timing and duration of water limitation during the plant life cycle as well as the distribution of water among deep versus shallow soil layers. As we discuss below, a better understanding of how osmotic adjustment is regulated would help both to answer these agronomic questions and to answer fundamental biological questions of how plants detect and respond to changes in water availability.

It must also be kept in mind that plant growth is not determined solely by physical limitations on turgor and water uptake. For example, when Arabidopsis seedlings are exposed to a moderately reduced ψ_w_ (−0.7 MPa) on PEG-agar plates we routinely observe that growth (quantified by fresh and dry weight or root elongation) is reduced by two-thirds compared with the unstressed high ψ_w_ control (Bhaskara et al., 2017; Longkumer et al., 2022). The data in Figure 2A make it clear that there is no sustained loss of turgor or tissue dehydration at −0.7 MPa that could explain such reduced growth. Rather the growth reduction observed is the result of active growth restriction in response to low ψ_w_ (Verslues and Longkumer, 2022). We can also hypothesize from these observations that a moderate low ψ_w_ treatment (such as −0.7 MPa, Figure 2A) would be ideal for identifying genotypes that either fail to restrict growth at low ψ_w_, and thus have increased growth maintenance compared with the wild-type, or genotypes that are more sensitive, perhaps because of a failure to osmotically adjust, and thus exhibit more severe dehydration and growth restriction compared with the wild-type. Indeed, our research has identified both negative and positive effectors of growth and osmotic adjustment (Verslues and Bray, 2004; Bhaskara et al., 2012; Longkumer et al., 2022).

A related, but more extensive, method of examining plant water relations often used by ecophysiologists is the generation of pressure–volume (PV) curves (Figure 2B; Koide et al., 1989). In this approach, a sample (typically a leaf, but could be a branch for larger plants or the entire shoot for smaller plants) is detached from the rest of the plant and is subjected to repeated bulk ψ_w_ and fresh weight measurements while being allowed to dehydrate. Fully hydrated weight is also measured to allow the RWC of the sample to be calculated at each of the ψ_w_ measurement points. This is referred to as a PV curve because traditionally the ψ_w_ was measured using a pressure chamber. However, recent refinements have shown that more rapid methods using a vapor pressure osmometer can also be effective (Bartlett et al., 2012a, 2012b;  Banks and Hirons, 2019). After performing a series of such measurements, a plot of −1/ψ_w_ versus RWC is constructed (Figure 2B). PV curves generally exhibit a steep initial nonlinear decline driven by a rapid drop in turgor (ψ_p_) until at a certain RWC the turgor pressure is lost (turgor loss point [TLP]). The linear decline in ψ_w_ past TLP is subsequently driven by the passive concentration of solutes with water loss. During the linear phase of the PV curve, the ψ_w_ will equal the osmotic potential (ψ_w_ = ψ_s_). Linear extension of this portion of the function can be used to estimate several parameters including the ψ_s_ at full hydration (−1/ψ_s100_), ψ_s_ at the point of turgor loss (−1/ψ_sTLP_) and RWC at the TLP (RWC_TLP_) (Figure 2B). Information about cell wall elasticity (ε, modulus of elasticity) can be derived from the slope of ψ_p_ between full hydration to the TLP: a steep slope (high ε) results from rigid cell walls while a shallow slope indicates elastic walls (low ε). Finally, estimates of the apoplastic water fraction (AWF) can be derived from the RWC at which ψ_w_ approaches infinity. The PV curve highlights how RWC can be difficult to interpret in the absence of other water relations data, especially whether ψ_w_ has decreased past the TLP. Without such data, it can be ambiguous whether a decreased RWC is associated with a reduction in turgor; or, whether turgor has already been lost and decreased RWC indicates dehydration of the tissue that is likely to damage cellular structure.

It has been proposed that ψ_s_ at the point of turgor loss (ψ_sTLP_) is a key determinant of plant adaptation to water-limited environments, as more negative values of ψ_sTLP_ extend the range of ψ_w_ over which the leaf can remain turgid and functional (Bartlett et al., 2012a, 2012b). Theoretically, plants may improve their drought tolerance by accumulating intracellular solutes (decrease ψ_s_) to decrease their TLP, decreasing intracellular volume while maintaining a relatively high amount of apoplastic water (increasing AWF), and increasing cell wall flexibility (decreasing ε) so that cell volume can decrease without a loss of turgor. Bartlett et al. (2012a, 2012b) provide a detailed discussion and examples of how various PV parameters may impact TLP. Also, the physiological literature contains reports of the water relations characteristics of most model or crop species. For example, a number of papers have reported water relations parameters for *A. thaliana* (Des Marais et al., 2012; Scoffoni et al., 2018), information that may be valuable for designing and interpreting Arabidopsis drought stress experiments.

Similar to the ψ_s_ and RWC data in Figure 2A, PV curve analysis (Figure 2B) can provide valuable baseline information for experimental design. For example, what is the range of ψ_w_ over which a plant is likely to retain the capacity to generate turgor and preserve cellular function? Studies employing ‘omics analyses, or other techniques, to understand how the plant acclimates and continues to function at low ψ_w_ would need to collect samples from tissue at ψ_w_ above the TLP. Conversely, we may expect that at ψ_w_ below the TLP where extensive cell shrinkage and cytorrhysis occur, many changes in gene expression or protein accumulation are likely to be involved in cellular damage control and can be interpreted in that light (Lang et al., 2014). A limitation of PV curve analysis has been that it is laborious and requires specialized pressure chamber equipment, although, as mentioned above, this limitation may no longer apply. Also, because a detached sample is used, PV curve analysis may not capture plastic responses of osmotic adjustment or cell wall properties as the plant acclimates to different moisture conditions over time. In this case, a series of samples would need to be analyzed from plants exposed to different moisture conditions to see if ψ_s100_ or ψ_sTLP_ are shifted as the plant acclimates to different environmental conditions (Bartlett et al., 2012a, 2012b). For instance, osmotic adjustment can be calculated with PV curves by subtracting the ψ_s100_ of drought-stressed plants from ψ_s100_ of well-watered plants (Figure 2C).

## Knowledge of plant water status can add a new level of insight to many types of physiological and molecular data

**Figure 3:**
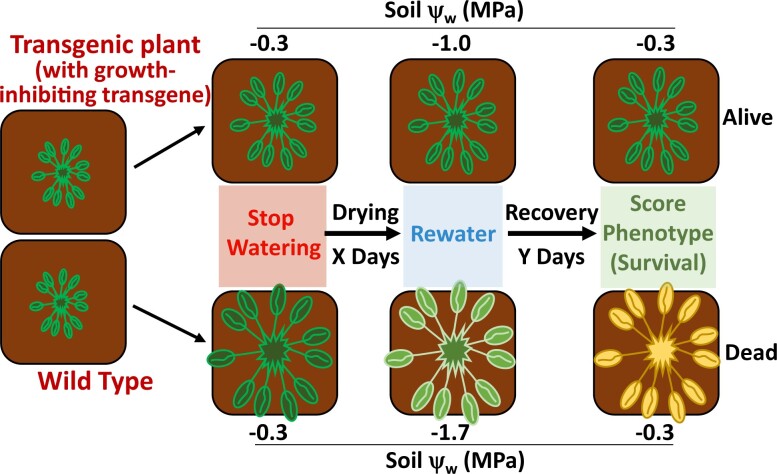
Potential pitfalls in uncontrolled soil drying experiments. The diagram depicts a scenario in which a reference genotype (wild-type) is compared with another genotype that is similar but has a slower growth rate resulting in a smaller plant size (less leaf area) at the start of the soil drying period (e.g. a mutant or transgenic line that has reduced growth compared with its wild-type background). If each genotype is grown in separate pots and subjected to a set period of soil drying, the smaller genotype will deplete the soil water more slowly by virtue of having less transpiring leaf area. In this case, the smaller genotype will likely have better recovery and less tissue damage after this set period of soil drying; however, this may not indicate a difference in drought resistance as the two genotypes were never exposed to the same severity of stress (the smaller genotype remained at higher ψ_w_). A more robust comparison of drought resistance between these two genotypes could be achieved by modifying the experiment design such that the soil water content (pot weight) is monitored and adjusted through the experiment to ensure that both genotypes experience the same ψ_w_. Alternatively (or in addition), both genotypes can be grown close together in the same symmetrical container such that they fully inter-root and thus experience the same soil ψ_w_ regardless of which genotype transpires more rapidly.

Interpretation of experimental data, including ‘omics data and various genetic analyses, can be greatly enhanced by knowledge of plant water status and ψ_w_ of the soil or growth media. Perhaps the most fundamental use of ψ_w_ and plant water relations data is to allow the experimenter to unambiguously tell the difference between low ψ_w_ avoidance versus tolerance. Many molecular genetic studies involve comparison of genetically modified plants (e.g. a mutant or transgenic line) to a wild-type control. A common experimental design is to grow the different genotypes in separate pots and subject them to a fixed duration of water withholding before phenotypic assay (often plant survival after re-watering; Figure 3). In this case, the severity of stress experienced by the plant is not controlled by the experimenter. Rather it is determined by the plant itself through the amount of water removed from the soil by transpiration. Plants that have less water loss through transpiration will deplete the soil water more slowly and thus be exposed to a less severe stress (higher ψ_w_) at the end of the water withholding period than plants with more rapid transpiration. Not surprisingly, the plants that were exposed to a less severe stress (higher ψ_w_) will typically have a higher survival rate at the end of the experiment. In the absence of measurements of soil ψ_w_ or water content, it is difficult, or impossible, to conclusively determine whether differences in survival are the result of differences in avoidance of water loss or differences in tolerance-related parameters. While both can be important, the underlying mechanisms are different.

A further complication is that mutants or transgenic lines that constitutively grow more slowly, often for reasons unrelated to stress response, can better survive the water withholding period solely by virtue of their relatively small transpiring leaf area. Such a difference is of uncertain value in terms of increasing plant productivity during drought. Similarly, unequal rates of soil water depletion can also complicate phenotypic analysis of genotypes that have altered ABA levels or altered sensitivity to ABA in stomatal closure. In uncontrolled soil drying experiments, these stomatal-dependent differences in soil water depletion will dominate the experimental results. If one wants to examine other, nonstomatal-related effects, steps need to be taken to ensure that all genotypes are exposed to the same soil ψ_w_. Similar concerns exist for comparisons among genotypes with altered stomal size or stomatal density. From a practical point of view for plant improvement, both avoidance of water depletion to conserve soil water and improve WUE (without sacrificing productivity in biomass gain or seed yield) as well as improved response to reduced ψ_w_ can all be of value. Which type of response may be most useful for plant improvement is a matter of debate and depends upon the timing, duration, and severity of drought in different environments. If genetic and molecular studies can do a better job of disentangling these two types of drought responses, we can provide more relevant information to develop new germplasm for use by agronomists who study crop productivity in the field.

The limitations of uncontrolled soil drying experiments and endpoint survival measurements have been highlighted in several studies. Skirycz et al. (2011) found that mutants reported to have increased survival after water withholding did not differ from the wild-type in growth responses to water limitation when they were exposed to an equal and moderate severity of soil drying using an automated pot weighing and watering system. Similarly, the dwarf mutant *chiquita1-1* (*chiq1-1*), also referred to as *constitutively stressed 1*, was originally described as drought “tolerant” based on uncontrolled soil drying survival assays (Bao et al., 2020). Later experiments where soil water content was monitored and controlled found that *chiq1-1* had reduced water usage because of its small size but did not differ in tolerance compared with the wild-type when both were exposed to the same severity of soil drying (Ginzburg et al., 2022). Another example of the avoidance of water loss phenomenon is provided in this issue by Wang et al. (2023), who describe a component in ABA signaling, SPIRAL1 (SPR1), that mediates microtubule disassembly during ABA-induced stomatal closure in Arabidopsis. When subjected to water withholding experiments, *spr1* mutant plants failed to close their stomata and therefore exhibited significantly greater water loss and lower survivability than the wild-type after a fixed time of water withholding. This indicates that SPR1 primarily affects drought avoidance. Such experiments do not themselves completely rule out additional effects of SPR1 on drought tolerance. However, determining whether or not SPR1 also affects microtubules in other cell types leading to differences in low ψ_w_ tolerance would require further experiments where *spr1* is exposed to the same external ψ_w_ and parameters, such as osmotic adjustment and RWC, and growth maintenance quantified.

Similarly, in cases where transcriptome or proteome data are collected at the end of uncontrolled soil drying experiments, it is difficult (or impossible) to deconvolute the effect of unequal soil drying from true genotype-dependent differences. This pitfall can be avoided by weighing pots and doing a partial re-watering to adjust all genotypes to the same soil water content or by growing the different genotypes together in a container that is sufficiently small and symmetrical to allow the plants to fully inter-root throughout the soil volume and thus be exposed to the same degree of drying even if different plants have differing rates of water usage (Verslues et al., 2006). At the same time, plants must be grown in a sufficient volume of soil to allow adequate root growth and prevent rapid drying that can obscure drought acclimation responses that occur over longer time scales as several days or longer are often needed for differences in growth, metabolism, or proteome remodeling to become apparent. As discussed above, consideration of the water holding capacity of the growth media can help in designing experimental conditions that allow the rate of soil drying and stability of the desired ψ_w_ treatment to be optimized (Dowd et al., 2019).

Many molecular stress studies focus on comparing the responses of well-watered control plants with plants that experience a single severity of drought stress. Unfortunately, stress treatments are often poorly controlled and therefore measurements collected from the stressed plants usually exhibit increased variability relative to measurements from control plants. It is simply easier to target a homogenous and benign soil ψ_w_ in a control treatment compared with consistently maintaining a specific soil ψ_w_ in treatment pots experiencing dynamic drying. This complicates the statistical analyses of stress experiments, often violating assumptions of homoscedasticity, and can reduce the power to observe real treatment impacts when they occur. Moreover, a single stress level may not capture the important range of response to the stress gradient plants would experience in nature. Many eco-physiological studies focus on exploring stress responses across a more dynamic and natural gradient of stress frequency and amplitude (Beier et al., 2012). These types of experiments are important as we anticipate that many stress responses result in nonlinear physiological or performance impacts. Measures of plant water status can also be used to evaluate relationships between physiological responses and the severity of water stress. For example, ψ_w_ measurements from leaves during the day can be a strong indicator of plant water stress as a function of soil water availability and atmospheric demand. This is because midday ψ_w_ reflects the balance of the amount of water supplied by the root system and transported through the xylem, and the strong demand caused by transpiration. Measures of plant ψ_w_-predawn are also valuable as a picture of water status in the absence of transpiration as stomates are generally closed at night. Predawn leaf ψ_w_ is expected to be in equilibrium with the “wettest” soil ψ_w_ accessed by roots (Richter, 1997) and should generally reflect the ψ_w_ of the soil prospected by the root system, although a number of factors can complicate this relationship (Donovan et al., 2001). Predawn ψ_w_ measures can therefore provide a whole-plant metric of the stress severity imposed by declining soil ψ_w_. Moreover, the difference in predawn ψ_w_ and ψ_w_ measured midday can give insight into the degree of maximum stress that plants experience due to the entire SPAC continuum, including transpiration loss from leaves (Martínez-Vilalta et al., 2014). Thus, measurements of plant ψ_w_ can be used to scale data to facilitate comparisons across different types of experiments and experimental conditions including plants grown in growth chambers versus greenhouse or field; or experiments using different types of growing media with differing soil water holding capacity; or comparisons among different species, cultivars, and genotypes.

**Figure 4:**
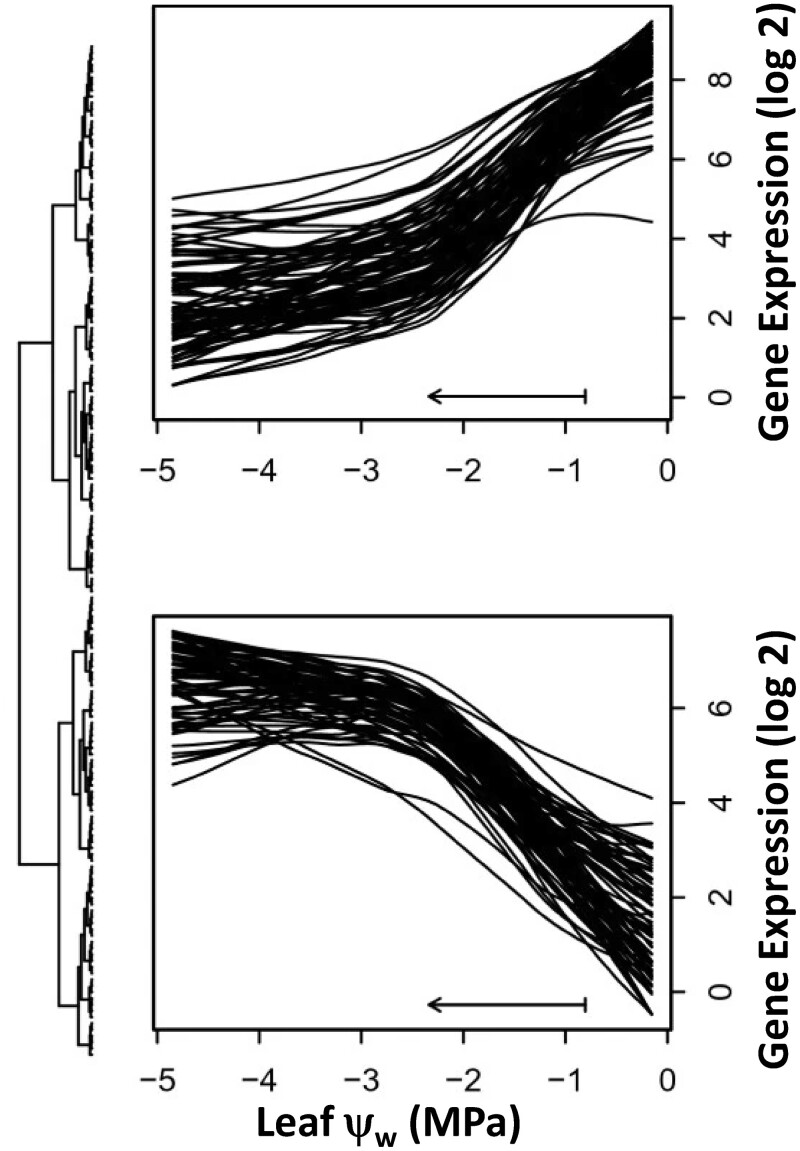
Nonlinear relationship between gene expression and predawn leaf ψ_w_ in a switchgrass drought experiment. Each line represents paired gene expression and physiological data from a progressive dry-down experiment with switchgrass. The sets of genes correspond to transcripts with significant nonlinear relationships with leaf ψ_w_. The fit lines for two clusters of stress-responsive genes indicate critical thresholds in expression that are likely related to turgor loss. Reprinted from Meyer et al. (2014).

From a biological perspective, using ψ_w_ measurements to identify genes that are differentially expressed can be more powerful and may give greater insight than only considering a contrast of a single stress treatment with a control. On a practical level, using actual ψ_w_ measurements to scale data can obviate the need to always “hit” a certain target level of stress when imposing the water limitation treatment. For example, Meyer et al. (2014) studied stress responses of switchgrass using a progressive dry-down experiment. The experiment generated predawn ψ_w_ measurements ranging from −4.8 to −0.6 MPa in the drought treatment and from −1.5 to −0.2 MPa in the controls. Analyses of covariation revealed nonlinear relationships between gene expression, predawn ψ_w_, and paired physiological traits (Figure 4) suggesting critical thresholds in drought stress responses that are likely associated with turgor loss. Similarly, Lovell et al. (2016) used ψ_w_-predawn and ψ_w_-midday to compare gene expression responses of switchgrass to soil drying in pots, field cylinders, and field rainout shelters to identify a core set of drought-responsive genes. Measuring ψ_w_-predawn not only allowed the three distinct experimental designs to be incorporated into a single analysis framework, it also allowed variability in stress generated from dry down rates (leading to differences in ψ_w_-predawn) to be incorporated in the analysis, thus increasing statistical power. This type of meta-analysis may allow more general discoveries of key biological processes involved in adaptive stress responses and recovery from water deficit.

We note that in this case, the use of ψ_w_ to scale the data is more robust than using RWC. This is because RWC does not directly measure the severity of the stress but rather represents a composite of the stress severity along with the plant response to stress in avoiding water loss and osmotic adjustment to retain water and turgor. Defining stress severity in terms of ψ_w_ allows the severity measurement to be independent of the plant’s stress response so that convolution of severity and response does not hamper data interpretation. That said, RWC measurements can be valuable in experiments conducted below the ψ_w-TLP_ as they give an indication of the extent of dehydration and the extent of cellular damage the plant has experienced.

## Genetic and genomic analyses are crucial to answer long-standing questions in plant water relations

Despite the well-developed methodology of plant water relations measurements illustrated above, and in contrast to drought avoidance responses, little is known about the genetic and cellular mechanisms that determine the capacity for osmotic adjustment or that determine cell wall properties that influence PV relationships in vegetative tissues (as opposed to guard cells which are distinct and not symplastically connected to other cells). Such mechanisms are not only important for drought research but also are a critical part of cell biology. For example, when external ψ_w_ does not change, cellular ψ_s_ remains constant even as cells transition from expansion (where rapid solute deposition is needed to drive water uptake) to cell maturation where cell expansion has ceased, and thus intracellular solute and water amounts are constant. The constancy of ψ_s_ and turgor during cell expansion and transition to elongation was demonstrated by Sharp et al. (1990) and Spollen and Sharp (1991) who found that even though low ψ_w_ increased solute content and decreased turgor overall, there was no change in these parameters as root cells exited the root elongation zone and ceased to expand.

In response to reduced external ψ_w_, the mechanisms that control cellular solute content must be altered to allow more solutes to accumulate. A decrease of cellular ψ_s_ by −0.5 MPa, which is within the capability of most plants, requires a 200 mM increase in solute content (assuming they act as ideal solutes). There is information about the regulation of individual proteins potentially related to water status, for example, aquaporins that control membrane water permeability (Ehlert et al., 2009; Sutka et al., 2011; Chaumont and Tyerman, 2014). However, the integrative mechanisms by which these molecular responses are coordinated to couple solute deposition with external ψ_w_ and solute dilution by cell expansion to maintain an appropriate ψ_s_ and turgor remain unknown. Interpretation of mutant or overexpression phenotypes is sometimes limited because effects on osmotic adjustment and plant water status were inferred rather than directly measured and avoidance versus tolerance effects may be convoluted (Osakabe et al., 2013; Um et al., 2018; Ren et al., 2021). Also, the solutes that accumulate differ between different compartments and these processes must somehow be coordinated (Wilson et al., 2014). This also illustrates how it is perhaps unlikely (although sometimes assumed) that changing the production or transport of a single solute is sufficient to change overall osmotic adjustment and water relations. For example, the stress signaling protein phosphatase highly ABA-induced1 (HAI1) has a greater effect on ψ_s_ than the closely related phosphatases HAI2 (also known as AIP1), ABA-insenstive1 (ABI1), or ABI2, even though mutants of all four phosphatases have increased proline accumulation compared with wild-type (Bhaskara et al., 2012). Similarly, cell wall responses to drought that can influence growth and PV relationships are varied and incompletely understood. There has been recent interest in how cell wall integrity affects cellular drought responses (see e.g. Bacete et al., 2022). Despite some recent progress, investigation of the genetic and cellular underpinnings of true tolerance of low ψ_w_ remains an underexplored area of drought research.

At least part of the reason for our limited understanding of the cellular basis of water relations and true drought tolerance mechanisms such as osmotic adjustment is that these phenotypes have seldom been the focus of molecular genetic studies. The studies mentioned above all started from the study of specific genes or metabolic pathways which may, or may not, affect core water relations parameters such as osmotic adjustment. Forward genetic or reverse genetic screening for drought tolerance traits has been surprisingly limited. In part, this is due to the difficulty in measuring such traits rapidly enough and in a nondestructive manner as well as by lack of reporters that directly respond to differences in solute content or ψ_w_. New types of sensors and techniques may help alleviate this bottleneck (see below). We anticipate that joining new high-throughput tools for measuring water status and ‘omics responses to stress will drive many new discoveries. For example, Condorelli et al. (2022) recently used genome wide association (GWA) studies of a Durum wheat diversity panel to identify candidate genes underlying osmotic adjustment.

## Measurement of plant water status and new methods used to scale up analysis of water status and physiological responses to drought

Plant water relations measurements are sometimes seen as laborious and require specialized equipment only available in a few laboratories. However, basic measurements of soil ψ_w_ or plant tissue ψ_w_ can be performed using readily available instruments (e.g. the WP4C) or several types of soil probes) for costs that are reasonable compared with the level of investment that is often required for ‘omic analyses whose interpretation would be enhanced by use of soil or plant ψ_w_ data. We think that the above examples convincingly show how much additional insight can be gained when water relations measurements are incorporated into the experimental design. Recent advances in techniques and instrumentation have made water relations measurements easier and more accessible. In one example, Sack and co-workers have described how ψ_sTLP_ can be determined more rapidly by using a vapor pressure osmometer (such as the widely available Wescor Vapro model) for direct estimation of ψ_s_ at full hydration (also referred to as π_0_; Bartlett et al., 2012a, 2012b;  Banks and Hirons, 2019).

Perhaps the biggest single change in instrumentation for drought research is the availability of automated weighing and watering systems. These systems allow individual pots to be weighed and rewatered up to predetermined soil water content to maintain plants under well-watered conditions or under a set severity of soil drying from mild stress to more severe stress. This can allow many plants to be exposed to the same severity of stress. Controlled soil drying can also be scaled to field studies using large soil monoliths and weighing lysimeters that allow gravimetric measures of evapotranspiration of plants from field soil (Schmidt et al., 2013). However, as described above, soil water content is not a parameter that can be used to report and reproduce the level of stress across laboratories because of different soil water holding capacities. Thus, the automated weighing and water approach can be coupled with the generation of soil moisture curves to relate soil water content to ψ_w_ for the soil type used (Figure 1B) and also potentially to PV curve analysis (Figure 2, B and C) to determine whether the stress imposed would be expected to push plants past the ψ_w-TLP_. As long as the same soil is consistently used, the soil moisture curves would only need to be generated once and could then be used to calibrate soil water content versus ψ_w_ for many subsequent experiments. A similar approach can be taken to incorporate the information in PV curves into the design of high-throughput experiments. This would allow the severity of stress imposed to be selected more precisely and reported in a manner that could be repeated in other laboratories, even if they are using a different type of soil. Thus, measurements of ψ_w_ are helpful at the experimental design stage both for reporting stress severity and for choosing the soil water content that imposes the desired severity of stress and also at later stages to improve interpretation of the resulting data.

Typically, automated weighing and watering combined with automated imaging to track plant growth parameters and hyperspectral cameras are increasingly being used to extract more data from such imaging analysis. Interestingly, data from hyperspectral imaging have been used to predict plant water relations parameters once proper calibration models were developed (Cotrozzi et al., 2020). Weighing and watering systems which track the amount of water added to each pot may also be used in calculations of gravimetric WUE, provided that nontranspirational soil drying is minimized. For those researchers without access to automated phenotyping systems, relatively simple procedures such as growing several genotypes together in one pot combined with manual pot weighing and watering along with checks of soil ψ_w_ can still allow robust measurements of growth responses to low ψ_w_ (see e.g. Bhaskara et al., 2017) and relatively simple procedures are available for medium throughput gravimetric WUE assays (Bhaskara et al., 2022). Agar plates incorporating high molecular weight PEG, when prepared properly, also allow medium throughput analysis of seedling low ψ_w_ responses while better mimicking the cytorrhytic type of water loss that plants experience during soil drying (Verslues et al., 2006).

Even more rapid detection of plant ψ_w_, perhaps even rapid enough for evaluations of large plant populations or to enable forward genetic screening for altered water relations, may become possible using new sensing technology. Jain et al. (2021) have described a hydrogel (which they named “AquaDust”) that reports leaf ψ_w_ based on changes in Förster Resonance Energy Transfer (FRET) between two fluorophores as the gel expands or contracts due to changes in hydration state. After infiltration into maize leaves, AquaDust FRET emission could be calibrated by using a pressure chamber to impose defined ψ_w_ onto the leaf. Postcalibration, AquaDust had sufficient resolution to detect ψ_w_ gradients along maize leaves. Cuevas-Velazquez et al. (2021) developed a genetically encoded FRET sensor which may detect osmotic changes inside living cells. They hypothesized that intrinsically disordered proteins, in their case an Arabidopsis late embryogenesis abundant (LEA) protein, may change conformation in response to changes in cellular osmolarity and this conformation change could be reported by the FRET signal between fluorophores attached to each of the protein. Changes in FRET were observed in response to large osmotic shifts in yeast, plant, or mammalian cells, but interestingly not in Arabidopsis which was the source of the LEA protein used to construct the sensor. Further testing and development of this technology will be of interest. In addition, several studies have reported the use of terahertz radiation to analyze tissue water content and construct PV curves (Baldacci et al., 2017; Browne et al., 2020; Li et al., 2020). Further development of all these tools is promising both to enable more extensive field measurements of plant water status and also to facilitate higher throughput laboratory screening.

## Conclusions

Plant biology is fundamentally intertwined with the study of plant–water relations, and yet the various fields of plant biology have historically taken disparate approaches to the analysis and reporting of plant water relations. Plant physiologists have traditionally studied parameters defining the relationship between the environment and plant–water status in exhaustive detail, but have yet to uncover many of the molecular or genetic processes that explain the diversity of traits and responses to water-deficit we see in nature. Ecologists often focus on larger temporal and spatial scales, evaluating how precipitation and water availability impact plant population or community dynamics, but these studies are often divorced from the physiological functions driving outcomes. Molecular and cell biologists usually simplify their experimental systems to afford greater control and precision, but in doing so handicap their ability to interpret or understand nature as it actually exists (Bergelson et al., 2021). Each of these perspectives has made valuable contributions to our understanding of plant function. Nevertheless, we argue that an integration of water relations data into cell and molecular studies is needed to truly gain an understanding of plant function and ultimately, to address the many impacts of climate change and ongoing threats to food security.

In this article, we have tried to show that converging on common and fundamentally sound ways of defining and reporting the severity of water limitation is not only advantageous for all types of drought researchers, it is also increasingly possible as water relations measurements continue to be refined and streamlined by new technologies and techniques, while genomic technologies also become ever more widely used. As a baseline for designing drought experiments, we would recommend that experiments seeking to compare the responses of multiple genotypes include sufficient data of plant or soil ψ_w_ to determine whether all the genotypes experienced the same decline in ψ_w_ during the stress period. This will allow a clear distinction of whether any differences in phenotype can be attributed to altered response to low ψ_w_ or altered water use such that some genotypes avoided water depletion and thus were not exposed to the same ψ_w_ as other genotypes. Also, as discussed above, using ψ_w_ to directly scale data can also enhance data interpretation, especially when combined with knowledge of related parameters such as the TLP. In this case, one can unambiguously determine if a loss of turgor, cellular dehydration, and damage have occurred versus moderate stresses where the plants can successfully acclimate to the reduced ψ_w_ and maintain cellular turgor and, at least partially, maintain growth. Applying these distinctions to large transcriptome or proteome data sets will help clarify damage responses versus acclimation responses to reduced ψ_w_. For higher throughput laboratory experiments with model plants such as Arabidopsis, there are well-established protocols for making plates of defined ψ_w_ severities that cover the range from mild stress to more severe low ψ_w_ (van der Weele et al., 2000; Verslues et al., 2006). As long as the protocols are followed (e.g. do not autoclave high molecular weight PEG), these experimental systems can apply stable ψ_w_ treatments (such that repeated checking of media ψ_w_ can be minimized) that mimic many of the key aspects of soil drying.

We also recommend a certain degree of circumspection in interpreting laboratory results. Drought is a complex phenomenon and the record of basic research in model organisms having an impact on improving drought resistance of crop plants or understanding the cellular basis for differences in the ecophysiology of drought-prone environments is not especially good. This is not because model organisms commonly studied are somehow flawed or lack drought resistance mechanisms. Rather, a key limitation is how we design experiments using these model organisms and how we interpret the data. There is much to learn as we still do not know either the genes and molecular mechanisms of how plants detect changes in water status, osmoregulate, and control turgor pressure, nor do we know what genetic factors will be most important to improve crop productivity (Nuccio et al., 2018) or understand ecosystem transformations as climate changes (Novick et al., 2022). Knowing your plants’ water status is fundamental to all these efforts.

## Notes

T.E.J and P.E.V designed the research, analyzed data, and both wrote the manuscript.

The authors responsible for distribution of materials integral to the findings presented in this article in accordance with the policy described in the Instructions for Authors (https://academic.oup.com/plcell) are: Thomas E. Juenger (tjuenger@austin.utexas.edu) and Paul E. Verslues (paulv@gate.sinica.edu.tw).
